# Auxin involvement in tepal senescence and abscission in *Lilium*: a tale of two lilies

**DOI:** 10.1093/jxb/eru451

**Published:** 2014-11-24

**Authors:** Lara Lombardi, Laia Arrom, Lorenzo Mariotti, Riccardo Battelli, Piero Picciarelli, Peter Kille, Tony Stead, Sergi Munné-Bosch, Hilary J. Rogers

**Affiliations:** ^1^Department of Biology, University of Pisa, Via Ghini 5, 56126 Pisa, Italy; ^2^Department of Plant Biology, Faculty of Biology, University of Barcelona, Avinguda Diagonal, 645, 08028 Barcelona, Spain; ^3^School of Biosciences, Cardiff University, Main Building, Park Place, Cardiff CF10 3AT, UK; ^4^Department of Agriculture, Food and Environment, University of Pisa, Via Mariscoglio 34, 56124 Pisa, Italy; ^5^School of Biological Sciences, Royal Holloway, University of London, Egham Hill, Egham, Surrey TW20 0EX, UK

**Keywords:** Abscission zone, ARF transcription factors, floral senescence, IAA, *Lilium longiflorum*, transcriptome.

## Abstract

Petal wilting and/or abscission terminates the life of the flower. However, how wilting and abscission are coordinated is not fully understood. There is wide variation in the extent to which petals wilt before abscission, even between cultivars of the same species. For example, tepals of *Lilium longiflorum* wilt substantially, while those of the closely related *Lilium longiflorum*×Asiatic hybrid (L.A.) abscise turgid. Furthermore, close comparison of petal death in these two *Lilium* genotypes shows that there is a dramatic fall in fresh weight/dry weight accompanied by a sharp increase in ion leakage in late senescent *L. longiflorum* tepals, neither of which occur in *Lilium* L.A. Despite these differences, a putative abscission zone was identified in both lilies, but while the detachment force was reduced to zero in *Lilium* L.A., wilting of the fused tepals in *L. longiflorum* occurred before abscission was complete. Abscission is often negatively regulated by auxin, and the possible role of auxin in regulating tepal abscission relative to wilting was tested in the two lilies. There was a dramatic increase in auxin levels with senescence in *L. longiflorum* but not in *Lilium* L.A. Fifty auxin-related genes were expressed in early senescent *L. longiflorum* tepals including 12 ARF-related genes. In *Arabidopsis*, several ARF genes are involved in the regulation of abscission. Expression of a homologous transcript to *Arabidopsis ARF7/19* was 8-fold higher during senescence in *L. longiflorum* compared with abscising *Lilium* L.A., suggesting a conserved role for auxin-regulated abscission in monocotyledonous ethylene-insensitive flowers.

## Summary

Strong evidence is presented for auxin regulating lily tepal abscission timing in relation to senescence. Transcriptome data was used to correlate auxin levels with expression of auxin-related genes.

## Introduction

Floral lifespan is tightly regulated and is species specific (Rogers, 2013). The ecological function of petals is in attracting pollinators, and hence petal lifespan is often tightly linked to pollination (van Doorn, 1997). However, even in the absence of pollination, petals have a limited lifespan that is terminated either by wilting or abscission. This can be further subdivided into species where petals are abscised fully turgid and those in which some wilting occurs first. In monocotyledonous plants, petals (or tepals) usually show some signs of wilting (McKenzie and Lovell, 1992; van Doorn and Stead, 1997). This ranges from *Hemerocallis* (daylily) in which 66% of dry weight (DW) is lost (Lay-Yee *et al.*, 1992) to *Alstroemeria* where only 20% is lost (Chanasut *et al.*, 2003). In many species, petal senescence is coordinated by the growth regulator ethylene; ethylene biosynthesis increases dramatically during petal senescence, and exposure to exogenous ethylene accelerates the process (van Doorn, 2001). However in an important group of flowers including the lilies, ethylene does not appear to play a major role in petal senescence (Rogers, 2013). A number of other plant growth regulators have been implicated in the regulation of floral senescence in ethylene-insensitive species (Arrom and Munné-Bosch, 2012*a*). In particular, auxin and cytokinin levels rose in *Lilium* L.A. var. ‘Courier’ prior to anthesis, falling thereafter (Arrom and Munné-Bosch, 2012*a*). Consistent with this, treatment of *Iris* cut flowers with cytokinins increased vase life slightly (van der Kop *et al.*, 2003), as did treatment of *Narcissus* (Hunter *et al.*, 2004) with gibberellic acid (GA_3_).

Abscission is a well-characterized developmental process, occurring in leaves, fruit, and floral organs. In cut flowers, it is an important factor in their post-harvest quality (van Doorn and Stead, 1997). In all abscising tissues studied, the process can be divided into four stages (Niederhuth *et al.*, 2013). The first stage involves the formation of an abscission zone (AZ) composed of a variable number of layers of small, cytoplasmically dense, cells. The structure of the AZ varies among species but is consistent within a single species (Taylor and Whitelaw, 2001). The timing of AZ formation also varies among species: for example, in tomato (Malayer and Guard, 1964) and *Arabidopsis* (Cai and Lashbrook, 2008) it forms long before abscission, while in cotton the AZ is formed only just before the organ is shed (Bornman *et al.*, 1967). Once formed, the AZ is competent to respond to abscission signals initiating the second stage of abscission. In flowers, these are only normally activated during senescence of the organ. However, in ethylene-sensitive flowers such as geranium, application of exogenous ethylene can result in very rapid petal abscission within 1–2h (Eversen *et al.*, 1993). This activation of the pre-formed AZ results in the third stage of abscission: specific degradation of middle lamellae between the AZ cells by the action of a suite of hydrolytic enzymes including polygalacturonases, cellulases, and endoglucanases (Roberts *et al.*, 2002). This allows the AZ cells to separate and the organ to abscise (Rogers and Stead, 2011). In the fourth stage, post-abscission, a protective layer forms over the site of abscission.

Auxin was identified as a key negative regulator of floral abscission over 50 years ago (Jacobs, 1962). This was demonstrated recently by manipulating auxin levels in AZ cells through activation of the bacterial auxin biosynthetic genes *iaaL* and *iaaM* specifically in these cells (Basu *et al.*, 2013). However, levels of auxin are also modulated by the balance between the free, active form, and conjugated inactive storage form (Ludwig-Müller, 2011). It was postulated that an influx of auxin into the AZ prevents abscission from taking place (Taylor and Whitelaw, 2001), making auxin a key regulator in the final decision to abscise. Auxin is synthesized in young tissues, and in *Arabidopsis* petals there is a transient increase in auxin levels in buds (Aloni *et al.*, 2006). The auxin is transported to other parts of the plant via a chemiosmotic mechanism mediated by PIN efflux carriers that determine the direction of flux (Leyser, 2006). In the leaf, it is generally accepted that the maintenance of constant polar indole-3-acetic acid (IAA) flux through the AZ prevents abscission (Osborne and Morgan, 1989; Taylor and Whitelaw, 2001; Roberts *et al.*, 2002). Polar auxin transport inhibitors, such as 1-*N*-naphthylphthalamic acid (NPA), provide useful tools for analysing the importance of auxin transport during developmental processes (Nemhauser *et al.*, 2000). The mode of action of NPA has not been fully elucidated, but *AtAPP1*, encoding a plasma-membrane metalloprotease, has been identified as a protein with a high affinity for NPA and a probable role in processing of PIN1 efflux carriers on the plasma membrane (Murphy *et al.*, 2002).

In *Arabidopsis*, cell-wall dissolution is modulated by auxin through the action of members of the auxin response factor (ARF) transcription factor family. ARF proteins are required for an auxin response: they bind to *cis*-elements in promoters of auxin-responsive genes resulting in their activation or repression (Ulmasov *et al.*, 1997). In *Arabidopsis*, there are 23 *ARF* genes (Wang *et al.*, 2007) and four of them have a role in organ abscission. *ARF1*, *ARF7*, and *ARF19* are directly upregulated by auxin, and these in turn upregulate *ARF2*, which acts to inhibit the expression of the hydrolytic enzymes (Ellis *et al.*, 2005) responsible for middle lamella breakdown. In rice, there are 25 ARF genes; *OsARF7* and *OsARF9* show the closest homology to *AtARF1* and *OsARF16* shows closest homology to *AtARF7* and *AtARF9*, while *OsARF4* is the closest homologue to *AtARF2* (Wang *et al.*, 2007).

*Lilium* species include commercially important cut flowers, especially as hybrids (Benschop *et al.*, 2010). The first group of hybrids produced were the Asiatic hybrids derived from species native to Central and East Asia. These have been further crossed to *Lilium longiflorum* to produce *L. longiflorum*×Asiatic (*Lilium* L.A.) hybrids. Lily hybrids include both abscising and non-abscising cultivars (van Doorn, 2001), although most cultivars show some wilting, with a longer time between wilting and tepal fall in Asiatic cultivars. The senescence patterns of both *L. longiflorum* (Battelli *et al.*, 2011) and the *Lilium* L.A. hybrid var ‘Courier’ (Arrom and Munné-Bosch, 2010, 2012*a*, *b*) have been recently studied. Tepals of *L. longiflorum* wilt substantially during senescence but remain attached, whereas tepals of *Lilium* L.A abscise following less severe wilting. This offers the opportunity to compare the senescence process in these two closely related genotypes. Specifically, the aim of this work was to test to what extent paradigms for the role of auxin in abscission, and upregulation of *ARF* genes developed with model species such as *Arabidopsis* can be applied to this taxonomically divergent, ethylene-insensitive genus.

## Materials and methods

### Plant material

*L. longiflorum* cv. ‘White Heaven’ was grown in a commercial greenhouse and *Lilium* L.A. var.’Courier’ (*L. longiflorum*×Asiatic hybrid) plants were obtained from greenhouse-grown bulbs. Individual flowers were harvested at the stage of closed bud by cutting above the last leaf. Flowers were placed in distilled water and kept in a growth chamber at 22 °C and 50% relative humidity. Under the conditions used, flower development and senescence progressed in a very predictable way from closed bud to full senescence (*L. longiflorum*) or abscission (*Lilium* L.A.).

Samples were collected from comparable developmental stages for the two species: closed bud (CB), full bloom flowers at anthesis (FB), flowers showing the first visible signs of senescence on outer tepals (early senescence, ES), and flowers at the end of their vase life (full senescence, FS), which was full dryness and wilting for *L. longiflorum* and abscission for *Lilium* L.A. Where appropriate, flowers were also harvested 1 or 2 d after either ES or FS.

### Exogenous treatments with IAA and NPA

Flowers at the stage of CB were treated with 10 µM IAA or with 50 µM NPA to inhibit auxin transport, and were added to the water in which the stems were immersed. Both treatments were applied throughout the experiment from CB to FS. Only outer tepals were sampled for all the analyses.

### Ion leakage

Discs (8mm diameter) were cut from each side of the central vein of the outer tepals about half way from the tip (20 discs per tepal) and placed in 10ml of distilled water in Petri dishes. After a 2h wash to remove ions from cut surfaces, the water was aspirated and fresh distilled water was added. Following incubation for 6h, the conductivity of the bathing solution (sample conductivity) was measured with a conductivity meter (HI-8733; HANNA Instruments). Fresh distilled water (10ml) was then added to the tepal discs and boiled for 15min. After cooling to room temperature, the conductivity was measured again to obtain the subtotal conductivity.

Ion leakage was expressed as relative conductivity, which was calculated as sample conductivity divided by total conductivity (the sum of sample conductivity and subtotal conductivity).

### RNA extraction and cDNA preparation

RNA was extracted from the outer tepals with TRI Reagent (Sigma, St Louis, MO, USA) according to the manufacturer’s instructions. RNA was subjected to DNase treatment using a TURBO DNA-free kit (Ambion, Austin, TX, USA) to remove contaminating genomic DNA. Two micrograms of RNA was reverse transcribed into cDNA using a High Capacity cDNA Reverse Transcription kit (Applied Biosystems, Foster City, CA, USA) in accordance with the manufacturer’s instructions.

### Illumina reference transcriptome sequencing of *L. longiflorum* tepals

Total RNA was extracted from the FB and ES stages of *L. longiflorum* tepals as described above and pooled. The quality of the RNA was checked by gel electrophoresis and capillary electrophoresis using a Bioanalyzer (RNA Nano Chip) and the concentration determined using absorption spectroscopy using a NanoDrop. A cDNA library was constructed by BaseClear (http://www.baseclear.com) following a Tru-Seq (Illumina) protocol and sequenced on an Illumina Hi-Seq 2000 using a 50-cycle paired-end protocol. A reference transcriptome was assembled using CLC Genomics Workbench (CLC bio). The validity of the assembly was performed by remapping (CLC Genomic Workbench) the original read onto the assembly and through functional annotation, with the former also providing a relative abundance of transcripts in the original sample. Functional annotation was performed using BLASTX and was used to interrogate the non-redundant protein sequence data downloaded from NCBI (1 November 2012) together with the Uniprot database (1 November 2012). Putative annotation assignment was performed at an E-value cut-off of <1E–05. BLAST results were integrated into BLAST2GO (Conesa and Götz, 2008), which was used to generate putative gene function and ontological assignments. Transcriptome data were deposited with the Sequence Read Archive (SRA) database at NCBI (SRA Experiment: SRX690392)

### Cloning of *Lilium* L.A. aminopeptidase P1 (APP1)

Degenerate primers were designed based on sequences from an *Alstroemeria* auxin-responsive gene *APP1* (Wagstaff *et al.*, 2010) and similar sequences from rice, *Ricinus*, and *Sorghum.* These were used to amplify a 195bp product. This was purified using a Qiaquick kit and ligated into pGEM-TEasy (Promega). Based on the sequence from the cloned fragment, specific primers were designed for quantitative real-time PCR (all primers are listed in Supplementary Table S1 at *JXB* online).

### Quantitative RT-PCR

Specific primers (Supplementary Table S1) were designed with Primer3 software (Rozen and Skaletsky, 2000) for the *Lilium APP1*, *ARF6/8*, *ARF 7/19*, and *AUX1*-like sequences derived from degenerate PCR or contigs from the *L. longiflorum* reference tepal transcriptome. PCR products from all the primer pairs were sequenced and compared with the available sequences to verify the specificity of the primers (sequences have been deposited in the EMBL data base under accession numbers LN606581, LN606582, LN606583, and LN606584). qPCR was carried out in a 7300 real-time PCR system (Applied Biosystems) using 50ng of cDNA and a SYBR^®^ Green PCR master mix (Applied Biosystems). The PCR product was further analysed by a dissociation curve programme from 95 to 60 °C. Expression of the 18S rRNA gene was used for internal normalization, using the PUV1 and PUV2 primers, which amplify a 226bp fragment (Dempster *et al.*, 1999). Data were analysed using the 2^–ΔΔCT^ method (Livak and Schmittgen, 2001) and are presented as the relative level of gene expression. All real-time qPCRs were run in triplicate with cDNAs synthesized from RNA extracted from three biological replicates.

### IAA extraction and analysis

Frozen tepal samples from control and NPA-treated flowers were homogenized in 5 vols of cold 80% (v/v) methanol and then stirred for 4h at 4 °C, before centrifugation at 2000*g* for 15min. The pellet was re-extracted twice; the supernatants were pooled and reduced to the aqueous phase under vacuum, and the pH of the supernatant was adjusted to 2.8 and partitioned four times against equal volumes of ethyl acetate. The samples were dried and dissolved in a small volume of 10% (v/v) aqueous acetonitrile containing 0.5% (v/v) acetic acid just before high-performance liquid chromatography. IAA was purified by high-performance liquid chromatography and quantified by gas chromatography/mass spectrometry as described previously (Mariotti *et al.*, 2011).

### Light microscopy

Abscission zones were excised from flowers at different stages of development. The pieces of tissue, approximately one-eighth of the cross-sectional area, were immersed in primary fixative comprising 3% glutaraldehyde, 4% formaldehyde in 0.1M PIPES buffer, pH 7.2, for a minimum of 1h. Specimens were then rinsed in 0.1M PIPES buffer, post-fixed in 1% buffered osmium tetroxide (1h), rinsed in buffer, block stained in 2% aqueous uranyl acetate (20min), dehydrated in an ethanol series, and embedded in Spurr resin (Agar Scientific, Stansted, UK) in the standard way. The polymerized blocks were then reoriented in order to be able to section perpendicular to the abscission zone. Semi-thin 0.5 µm sections were cut on a Leica OMU 3 ultramicrotome and stained with 1% toluidine blue in 1% borax and photographed on a light microscope using a Nikon Coolpix 4500 digital camera.

### Detachment force

For *L. longiflorum*, the free portion of the tepals was removed and the filaments and ovary trimmed further to ensure that when clamped only the corolla tissue was held. The clamp, with attached flower, was connected to a strain gauge (M/no DFG-1K; Shimpo), the pedicel was grasped firmly, and a single straight pull was employed to remove the corolla. Where the corolla tore, or was pulled from the clamp, the break strength was recorded as ‘in excess of the recorded value’; hence, for some stages the values presented in Fig. 3B and C are underestimates of the force needed to detach the corolla. For *Lilium* L.A., individual tepals were trimmed by about one-third of their length, and then clamped and the detachment force determined using a single straight pull of the pedicel. The process then was repeated for the remaining tepals of that flower.

## Results

### Senescence markers show significant differences in *L. longiflorum* and *Lilium* L.A.

**Fig. 1.**
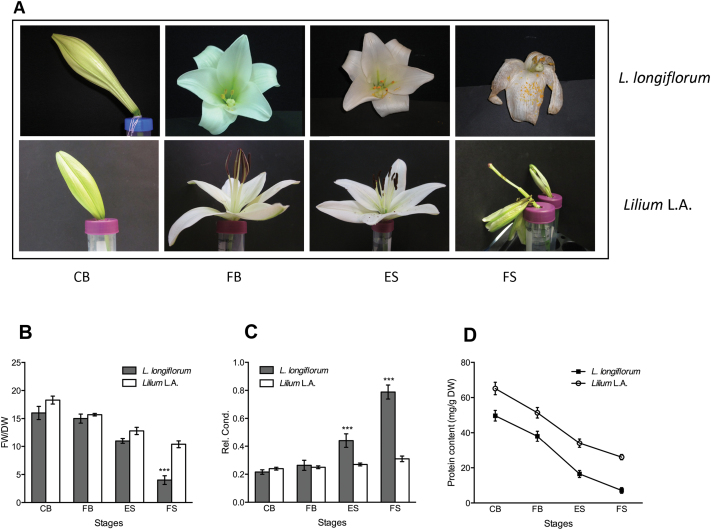
Floral senescence progression in *L. longiflorum* and *Lilium* L.A. (A) Equivalent stages based on floral development and signs of visible senescence defined by Battelli *et al.* (2011) and Arrom *et al.* (2012). CB, closed bud; FB, full bloom; ES, early senescence; FS, full senescence. (B, C) Changes in FW/DW (B) ion leakage (C), and protein content (D) with senescence in the outer tepals of the two genotypes. Results are means±standard error (SE), *n*=20. Asterisks indicate significant differences between the two genotypes at each stage as determined by Student’s *t*-test: ***P<0.001.

Although flower life progresses through similar stages in *L. longiflorum* and *Lilium* L.A., the final destiny of the tepals diverged. In *L. longiflorum*, tepals wilted substantially during senescence with visible browning and dehydration; however, they remained attached to the flower. In contrast, *Lilium* L.A. tepals abscised without wilting while still relatively turgid (Fig. 1A). Tepal fresh weight (FW), DW, ion leakage, and protein content were compared between *L. longiflorum* and *Lilium* L.A. to establish first a benchmarking of the senescence progression between the two flowers and then to determine key differences that might be related to the different strategies of wilting versus abscission in these closely related genotypes. ES was defined in both genotypes as the earliest stage showing visible signs of senescence such as tepal browning and increased translucency, while FS was defined in *Lilium* L.A. as the time of abscission and in *L. longiflorum* as complete wilting and full dehydration of the tepals (Fig. 1A). In both genotypes, FW/DW fell as the tepals progressed from CB through FB to senescence (Fig. 1B). However, in the later stages of senescence, there was a marked difference. Whereas in *Lilium* L.A. tepals there was a gradual reduction in FW/DW, in *L. longiflorum* there was a sharp decline between the early and late senescence stages. There were also marked differences in the pattern of ion leakage (Fig. 1C). In *Lilium* L.A., electrolyte leakage remained relatively low and constant throughout bud opening and senescence, while in *L. longiflorum* there was a sharp increase in ion leakage between early and late senescence. In contrast, the decline in protein levels followed essentially the same trend in the two genotypes, although protein levels in *L. longiflorum* were lower throughout (Fig. 1D).

### An AZ forms in both *L. longiflorum* and *Lilium* L.A., but the detachment of *L. longiflorum* is anomalous

**Fig. 2.**
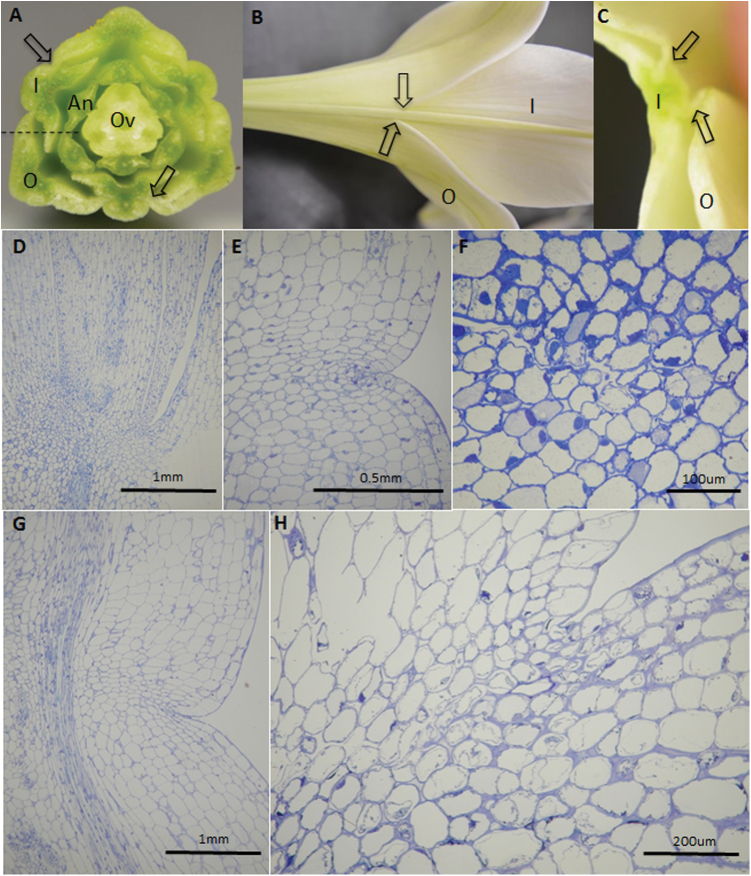
The AZ in *L. longiflorum* and *Lilium* L.A. (A–C) Transverse section across a young flower of *L. longiflorum* ‘White Heaven’ (A) showing the central ovary (Ov), anthers (An), and inner (I) and outer (O) tepals. The margins of the outer tepals are fused with the midrib of the inner tepals (arrows), which are shown on the outside in (B) and at higher magnification in (C). (D–F) Longitudinal section through the corolla base of *L. longiflorum*, at FB (D, E) and at ES (F). (G, H) Longitudinal section through the corolla base of *Lilium* L.A. at FB (G) and at ES (H).

In *L. longiflorum*, the outer tepals and inner tepals are fused over the lower third of the tepal length, with the margins of the outer tepals fused to the midrib of the inner tepals. Thus, in transverse section (Fig. 2A–C), there can appear to be two whorls of tepals and, if looking even closer to the pedicel, the individual outer tepals may also overlap giving the appearance of multiple whorls of tepals. When examining the AZ in longitudinal section, this results in several structures being visible, each with its own putative AZ (Fig. 2D).

In section, the AZ of each *L. longiflorum* tepal was visible. Even in freshly opened flowers at FB (Fig. 2D), the putative AZ, comprising a series of smaller cells, could be recognized. At higher magnification, the beginning of a fracture line was discernible running between these cells (Fig. 2E). However, in older flowers, several days after FS, this fracture line had not progressed significantly (Fig. 2F). The AZ was identifiable at the base of *Lilium* L.A. tepals even in CBs (Fig. 2G), and cell-wall dissolution was visible in the AZ of those flowers approaching ES (Fig. 2H).

**Fig. 3.**
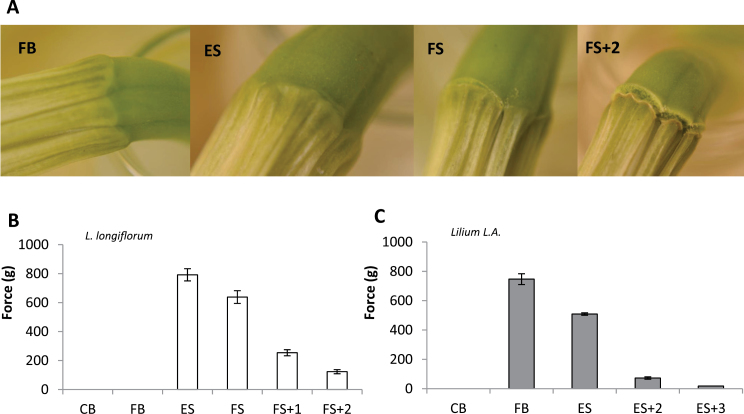
Detachment of tepals in the two lily genotypes. (A) Outside of the corolla base of *L. longiflorum* at FB, ES, FS, and beyond. (B, C) Force (*g*) required to remove the corolla of *L. longiflorum* (B) at each stage (the force required at CB and FB could not be determined as the corolla tissue tore) plus at 1 and 2 d following the FS stage, and in *Lilium* L.A. (C) (again detachability could not be determined at CB and the value for FB was determined only from those that detached and may represent a considerable underestimate as *n*<10; in *Lilium* L.A., by FS the tepals had abscised naturally. Values are means±SE with *n*≥10 unless otherwise stated.

Detachment force was measured in both lilies to establish whether the AZs observed were fully functional. In *L. longiflorum*, small dark lines, which later developed into cracks (Fig. 3A), were visible on the outside of the tissue at the point where the AZ would be expected to be situated. The fusion of the tepals in *L. longiflorum* meant that, unlike in *Lilium* L.A., only the force to detach the corolla, not individual tepals, could be quantified (Fig. 3B, C).

In early-stage buds through to freshly opened flowers, the force to remove the corolla in *L. longiflorum* either exceeded 1000*g* (the limit of the force gauge) or more often the corolla tissue tore at approximately 700–800*g*, although in some fully open flowers detachment of the corolla at the AZ occurred at 500–600*g* (Fig. 3C). As the flowers senesced and wilted, the extent of the cracking (Fig. 3A) increased, concomitant with a decrease in the force needed to detach the corolla. However, in flowers with wilted, browning tepals (i.e. beyond FS) where the external cracks appeared to be both extensive and deep (FS+2 in Fig. 3A), a force of 100–200g was still required to remove the corolla (Fig. 3B), showing that the abscission process was far from complete, even in senescent flowers that had passed the end of their vase life. In younger, freshly opened flowers (FB), no external cracking was visible (Fig. 3A) and the corolla could not be forcibly be removed by the strain gauge (Fig. 3B).

In *Lilium* L.A., some tepals could be detached from flowers that appeared to be at the FB stage, although most tore rather than detached (Fig. 3C). By the ES stage, virtually all tepals could be detached, and the force needed was greatly reduced (~600*g*); by the FS stage, all tepals had already abscised, but in the time between ES and FS the force needed to detach the tepals decreased, as would be expected for an organ showing a typical abscission process (Fig. 3C).

### Auxin levels differ between the two lilies during tepal senescence

**Fig. 4.**
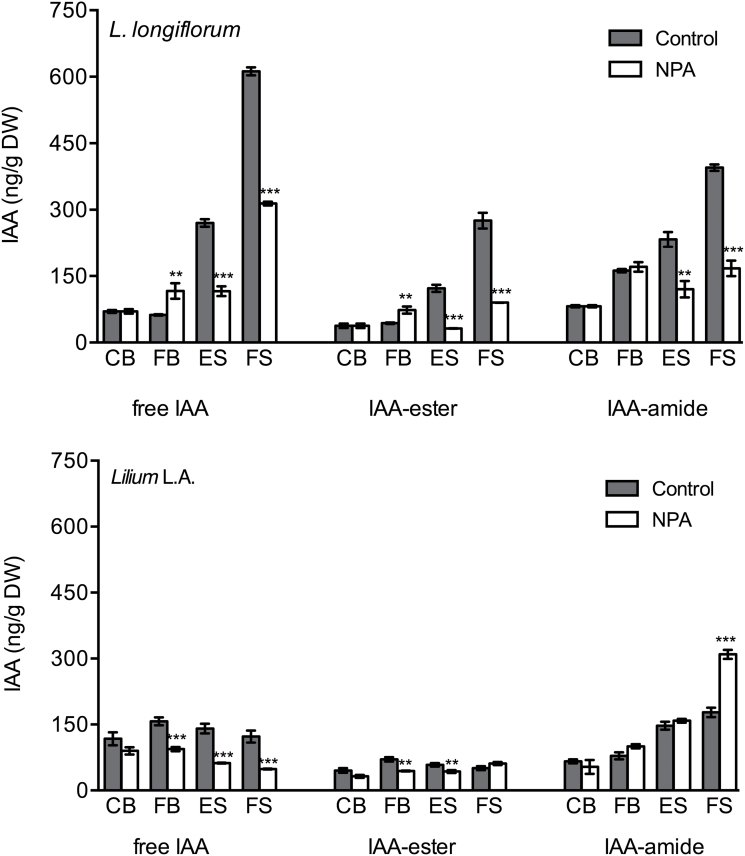
Concentrations of endogenous free and conjugated IAA in the outer tepals of control and NPA-treated (50 µM) flowers. (A) *L. longiflorum* and (B) *Lilium* L.A. at stages defined in Fig. 1. Results are means±SE, *n*=10. Asterisks indicate significant differences between the two genotypes at each stage as determined by Student’s *t*-test: **P<0.05; ***P<0.001.

As auxin appears to be a key regulator of abscission, the level of total IAA as well as active free IAA and inactive conjugated forms (ester-linked to sugars and amide-linked to amino acids and peptides) was determined for both lily genotypes. Around the time of flower opening (from CB to FB), IAA content was similar in the two lilies, at about 100–150ng g^–1^ of DW. Then, as senescence progressed, in *L. longiflorum* both free IAA and conjugated IAA increased dramatically (Fig. 4A), although the ratio of free to conjugated IAA was essentially 1:1 throughout the flower lifespan (Supplementary Fig. S1 at *JXB* online). In contrast, free IAA levels in *Lilium* L.A. remained low at every stage, from CB to abscission, while the portion of inactive IAA-amide gradually increased (Fig. 4B). Note that in *Lilium* L.A. the ratio of free to conjugated IAA changed from about 1:1 to 1:2 between CB and abscission (Supplementary Fig. S1).

The effect of exogenous application of IAA was tested on both lily genotypes. No effect was seen either on senescence progression or on timing of abscission (data not shown).

### Analysis of the lily tepal transcriptome reveals 50 genes related to auxin biosynthesis and perception including 12 ARF-like genes.

Forty million reads of a *L. longiflorum* reference transcriptome from ES/FB tepals were assembled into 13 000 unigenes. Fifty unigenes showed homology to auxin-related genes from other species (Table 1) consistent with the high levels of auxin detected. Twelve unigenes showed homology to genes belonging to the ARF family of transcription factors, and could be assigned to six *Arabidopsis* homology groups: *AtARF6/8*, *AtARF9/18/11*, *AtARF19*, *AtARF3/4*, *AtARF7/19* and *AtARF16* (Supplementary Table S2 at *JXB* online). Homology was assessed based on the inclusion in the contig sequence of motifs III and IV, which are consensus sequences shared by AUX/IAA proteins but are discriminatory between different *ARF* genes in rice (Wang *et al.*, 2007) (Supplementary Fig. S2 at *JXB* online). Primers were designed to two contigs with homology to *AtARF6/8* and *AtARF7/19* (contigs 651 and 7111, respectively) and were verified by PCR and sequencing of products.

**Table 1.** L. longiflorum petal unigenes showing homology to genes with auxin-related functions

Lily contig	Accession code	Match on nr database	E-value
651	A2YG67	Auxin response factor 17 *Oryza sativa*	0
1348	Q653U3	Auxin response factor 17 *Oryza sativa*	6E–11
1628	Q9XED8	Auxin response factor 9 *Arabidopsis thaliana*	3E–32
2468	Q9XED8	Auxin response factor 9 *Arabidopsis thaliana*	3E–83
5803	Q6YZW0	Auxin response factor 21 *Oryza sativa*	2E–10
6507	Q9ZTX9	Auxin response factor 4 *Arabidopsis thaliana*	2E–28
7111	Q0D9R7	Auxin response factor 19 *Oryza sativa*	5E–60
8123	Q0DGS1	Auxin response factor 14 *Oryza sativa*	6E–16
9023	Q9ZPY6	Auxin response factor 11 *Arabidopsis thaliana*	3E–13
10581	Q653U3	Auxin response factor 17 *Oryza sativa*	3E–12
11452	Q5JK20	Auxin response factor 4 *Oryza sativa*	4E–25
11651	Q653H7	Auxin response factor *Oryza sativa*	3E–32
2713	B9G2A8	Auxin transport protein BIG *Oryza sativa*	0
3454	Q96247	Auxin transporter protein 1 *Arabidopsis thaliana*	1E–115
7797	Q9FEL6	Auxin transporter-like protein 3 *Medicago truncatula*	3E–83
6782	Q5SMQ9	Auxin efflux carrier component *Oryza sativa*	9E–12
4681	Q94BT2	Auxin-induced in root cultures *Arabidopsis thaliana*	2E–28
5083	Q6J163	Auxin-induced protein 5NG4 *Pinus taeda*	2E–23
10928	Q6J163	Auxin-induced protein 5NG4 *Pinus taeda*	5E–10
32	Q6J163	Auxin-induced protein 5NG4 *Pinus taeda*	3E–21
9356	Q6J163	Auxin-induced protein 5NG4 *Pinus taeda*	2E–31
4137	Q6J163	Auxin-induced protein 5NG4 *Pinus taeda*	9E–34
3596	Q6J163	Auxin-induced protein 5NG4 *Pinus taeda*	3E–35
3456	Q6J163	Auxin-induced protein 5NG4 *Pinus taeda*	6E–46
1865	Q6J163	Auxin-induced protein 5NG4 *Pinus taeda*	3E–52
1341	Q6J163	Auxin-induced protein 5NG4 *Pinus taeda*	1E–57
5697	Q6J163	Auxin-induced protein 5NG4 *Pinus taeda*	1E–77
7896	P33083	Auxin-induced protein 6B *Glycine max*	3E–09
5806	P40691	Auxin-induced protein PCNT115 *Nicotiana tabacum*	3E–20
1364	P40691	Auxin-induced protein PCNT115 *Nicotiana tabacum*	6E–65
949	Q05349	Auxin-repressed 12.5kDa protein *Fragaria ananassa*	2E–16
7600	Q5VRD1	Auxin-responsive protein IAA1 *Oryza sativa*	8E–21
2243	Q5VRD1	Auxin-responsive protein IAA1 *Oryza sativa*	5E–43
4986	Q6AT10	Auxin-responsive protein IAA15 *Oryza sativa*	2E–17
2817	Q5Z749	Auxin-responsive protein IAA21 *Oryza sativa*	2E–33
3836	Q9ZSY8	Auxin-responsive protein IAA27 *Arabidopsis thaliana*	9E–33
8767	P0C132	Auxin-responsive protein IAA30 *Oryza sativa*	5E–10
3951	P0C132	Auxin-responsive protein IAA30 *Oryza sativa*	1E–42
1293	Q6H543	Auxin-responsive protein IAA7 *Oryza sativa*	4E–21
3646	P32295	IAA-induced protein ARG7 *Vigna radiata*	0.000002
5808	P32295	IAA -induced protein ARG7 *Vigna radiata*	7E–07
2930	P32295	IAA -induced protein ARG7 *Vigna radiata*	3E–09
2438	Q67UL3	Probable auxin efflux carrier component 1c *Oryza sativa*	5E–82
3338	Q9LW29	AUXIN SIGNALING F-BOX 2 *Arabidopsis thaliana*	0.000006
9124	Q9LW29	AUXIN SIGNALING F-BOX 2 *Arabidopsis thaliana*	0.000009
12877	Q9LW29	AUXIN SIGNALING F-BOX 2 *Arabidopsis thaliana*	2E–11
11286	Q9LW29	AUXIN SIGNALING F-BOX 2 *Arabidopsis thaliana*	9E–12
1440	Q9LW29	AUXIN SIGNALING F-BOX 2 *Arabidopsis thaliana*	1E–171
12808	Q9LPW7	AUXIN SIGNALING F-BOX 3 *Arabidopsis thaliana*	5E–26
7557	Q0DKP3	Transport inhibitor response 1-like protein *Oryza sativa*	1E–55

**Fig. 5.**
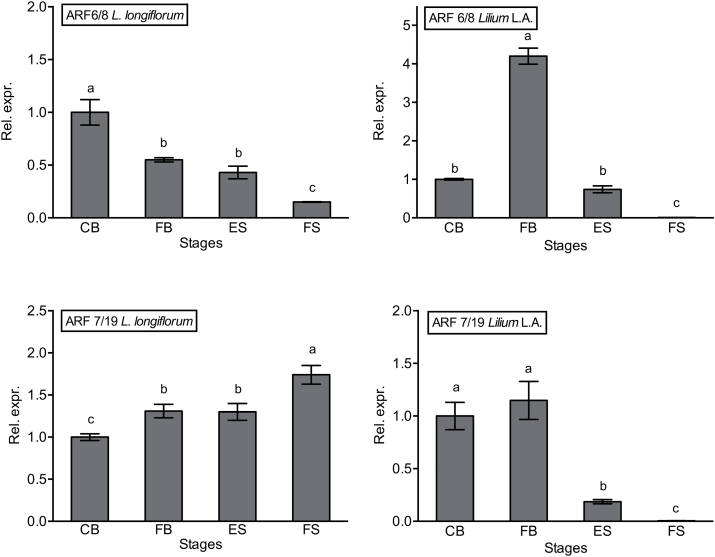
Relative expression of *ARF*-like genes by real time RT-PCR. Transcript levels of the *ARF6/8*-like gene (A, B) and the *ARF7/19*-like gene (C, D) in *L. longiflorum* (A, C) and *Lilium* L.A. (B, D) at the stages defined in Fig 1. Results are means±SE, *n*=10. Different letters indicate significant differences among stages as determined by one-way analysis of variance.

The transcript level of the *ARF6/8* homologue fell with tepal age in both lilies but with different patterns (Fig. 5A, B). In *Lilium* L.A., there was a peak in transcript level at FB, which then essentially disappeared at abscission; in *L. longiflorum*, the highest expression was in CB and expression then declined gradually.

The transcript level of the homologue of *Arabidopsis ARF7/19* fell with development and senescence in *Lilium* L.A., while in *L. longiflorum*, which has the higher auxin content and delayed incomplete abscission, levels rose slightly during flower life and were much higher than in *Lilium* L.A. at both early and late stages of senescence (ES and FS) (Fig. 5C, D).

### NPA has opposite effects in *L. longiflorum* and *Lilium* L.A.

When the flowers at stage CB were treated with NPA, a widely used auxin transport inhibitor, senescence progression in *L. longiflorum* and the time of abscission in *Lilium* L.A. did not change (data not shown). However, NPA induced remarkable changes in IAA concentration in the whole tepal (Fig. 4). In both genotypes, the levels of free IAA during the senescence stages were reduced by more than 50%. The effect of NPA treatment on the conjugated IAA pool differed substantially between the two genotypes. In *L. longiflorum*, IAA-ester and IAA-amide were reduced to about 30% of the control during early and late senescence. In contrast, in *Lilium* L.A., IAA-amide showed a dramatic increase at the time of abscission and IAA-ester levels did not change significantly.

The overall result was that, at the last stage of flower life, NPA treatment had the opposite effect on total IAA amount in the tepal in the two genotypes. In fact, in *L. longiflorum*, there was an almost 2-fold reduction in total IAA concentration, while in the abscised tepals of *Lilium* L.A., total IAA actually increased slightly, mainly due to the rise in IAA conjugates (Supplementary Fig. S1). Consequently, the ratio of free to conjugated IAA went from 1:1 to 1:7 at the time of abscission.

To determine whether the distribution of auxin across the tepal differed between genotypes, IAA content in the different regions of the tepal was determined at the FS stage. Free IAA was not distributed equally along the tepal axis. In both genotypes, the IAA concentration was higher at the base compared with the tip (Table 2) with levels over 4-fold higher in *L. longiflorum* in each region. NPA treatment resulted in a fall in IAA concentration at the tip and a greater decrease in the middle section but a significant increase at the base of the tepal in both genotypes.

**Table 2.** Free IAA content across petals of the two genotypes at FSLetters indicate a significant differences between control and treatment (*P*<0.05).

IAA (ng g^–1^ DW)	*L. Longiflorum*		*Lilium* L.A.	
	Control	NPA	Control	NPA
Tip	341±9 a	262±7 b	55±0.2 a	40±0.8 b
Middle	612±8.8 a	314±4.3 b	122±3.5 a	48±0.01 b
Base	504±3.6 a	572±11 b	125±1.3 a	140±0.9 b

### APP1 and AUX1 gene expression

To investigate further the role of auxin transport, expression of *APP1*, which is involved in the processing of PIN efflux carriers, and *AUX1*, an auxin influx transporter, was determined in the two lilies during tepal development and senescence.

**Fig. 6.**
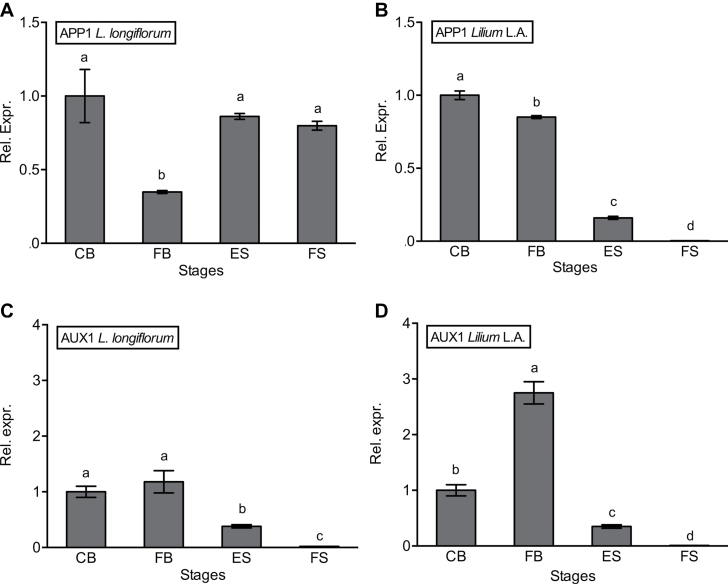
Relative expression of *APP1-*like and *AUX1*-like genes by real-time RT-PCR. Transcript levels in *L. longiflorum* (A, C) and in *Lilium* L.A. (B, D) at the stages defined in Fig 1. Results are means±SE, *n*=10. Different letters indicate significant differences among stages as determined by one-way analysis of variance.

Using degenerate primers designed to sequences from *Alstroemeria*, rice, *Ricinus*, and *Sorghum*, a 195bp fragment homologous to *Arabidopsis AtAPP1* was obtained from *Lilium* L.A. tepal cDNA (Supplementary Fig. S3 at *JXB* online). Quantitative expression analysis showed that, in both lilies, *APP1* transcript levels were highest in the CB, but then there was an opposite trend during senescence (Fig. 6A, B). Expression was undetectable at abscission in *Lilium* L.A. while it was still high at the last stage of senescence in *L. longiflorum*, coincident with the highest levels of IAA (Fig. 3A).

The expression of the homologue of *AUX1* decreased with progression of senescence in both lilies (Fig. 6C, D), although levels were almost 3-fold higher at FB in *Lilium* L.A. Thus, ES in *Lilium* L.A. was accompanied by a 2-fold greater fall in expression compared with *L. longiflorum*.

## Discussion

Given the differences in senescence progression and final abscission between the two lily genotypes, the focus of this work was to discover whether contrasting auxin levels could explain the differences in progression of abscission in relation to senescence.

First, a comparison was made of the senescence patterns between the two genotypes: *L. longiflorum*, where petals remain attached and wilt, and *Lilium* L.A., where tepals abscise turgid. Visual similarities between the developmental stages from CB to ES were supported by measurements of FW/DW and ion leakage. In both genotypes, FW/DW fell gradually and ion leakage remained constant from the CB to the FB stage. However, as the flowers entered senescence, their programmes diverged. Whereas in *Lilium* L.A. FW/DW continued to fall gradually, there was a sudden 2-fold drop between early and late senescence stages in *L. longiflorum*. This was accompanied by a doubling in ion leakage. These parameters are indicative of a rapid increase in water loss and cell death in *L. longiflorum* as senescence progresses but not in *Lilium* L.A. This is common in many species, including other lilies such as *Hemerocallis* (Lay-Yee *et al.*, 1992), where there is substantial weight loss during senescence. In *Alstroemeria*, as in *L. longiflorum*, ion leakage rose, sharply coincident with the first signs of visual senescence (Leverentz *et al.*, 2002). The lack of increases in the rate of weight loss and ion leakage in *Lilium* L.A. are reminiscent of other species such as tulip where FW fell to only about 70% of its maximal value (Azad *et al.*, 2008), and *Prunus yedoensis* petals where no signs of PCD were detected prior to turgid abscission (Yamada *et al.*, 2007). Thus, not only do *Lilium* L.A. tepals abscise but they do so in a relatively intact state, suggesting a very different senescence programme to *L. longiflorum*.

One possibility for the lack of abscission in *L. longiflorum* would be the failure to develop an AZ at all (Rogers and Stead, 2011). However, examination of the base of the outer tepals of both genotypes revealed that there is what appears to be a functional AZ in both. This suggests that the failure to completely abscise *L. longiflorum* tepals must depend on the signals that control the timing and completion of abscission. Differences in auxin levels in the AZ are a key component in tipping the balance towards abscission (Taylor and Whitelaw, 2001). Therefore, levels of auxin were compared throughout development and senescence across whole tepals of the two lilies. The dramatic increase in auxin levels in late senescence in *L. longiflorum* tepals would be consistent with a role for this growth regulator in delaying activation and/or completion of the AZ while senescence processes such as cell death and water loss are still progressing in other cells or tissues. The constant and low levels of auxin in *Lilium* L.A. are consistent with an earlier activation and completion of the AZ, allowing tepals to abscise turgid. Since the balance between free and conjugated auxin is also important in determining the activity of this growth regulator (Rosquete *et al.*, 2012), the finding that at FS *L. longiflorum* tepals contained higher levels of free compared with the two inactive conjugated forms further indicates that the auxin is active in this tissue. In contrast, in *Lilium* L.A. tepals, there was substantially more IAA-amide at FS than free IAA, indicating that, as well as lower levels of auxin, more of it was also conjugated and therefore inactive. This suggests several possible regulatory methods: an increased biosynthesis or transport, or reduced metabolism of the auxin, as well as differences in the activity of enzymes regulating the balance between conjugated IAA and free IAA.

To understand further the role of auxin in these lilies, auxin-related genes were derived from an early senescent tepal reference transcriptome. Target sequences for lily *ARF* genes known in *Arabidopsis* to be important in regulating abscission were sought: specifically *Arabidopsis ARF1*, *ARF7/19*, and *ARF2*. No homologues to *ARF1* or *ARF2* were obtained, despite a good depth of sequencing. This could be due to low levels of expression at this stage of development: in *Arabidopsis*, *ARF1* is expressed at very low levels during petal and leaf senescence, and *ARF2* is upregulated in senescent leaves but not in older petals (eFP browser; Winter *et al.*, 2007). However, putative homologues of *ARF7/19* were identified, and their expression pattern in *L. longiflorum* during senescence was consistent with upregulation of the expression of these genes in *Arabidopsis* older petals. In *Arabidopsis*, *ARF19* is induced by auxin (Wilmoth *et al.*, 2005) and is part of a positive-feedback loop involving *ARF7*. Thus, the upregulation of the lily *ARF7/19*-like gene is also consistent with an increase in auxin in this genotype. In contrast, the fall in expression of the *ARF7/19*-like gene with senescence in *Lilium* L.A. is consistent with the slight fall in free auxin in this genotype and also with the completion of abscission. As a comparison, the expression of *ARF6/8* was also analysed and found to be downregulated during senescence in both lily genotypes. In *Arabidopsis*, *ARF8* is involved in petal growth and expansion (Varaud *et al.*, 2011), and *ARF6* and *ARF8* are strongly expressed in both young and older petals, but their expression declines with petal age.

Having established a clear correlation between auxin levels and abscission timing in the two lily genotypes, we also asked if auxin transport was involved, since this may be more important that absolute concentration of IAA. NPA treatment did not affect the timing of senescence in *L. longiflorum* or abscission in *Lilium* L.A. This is in contrast to leaf abscission in *Mirabilis jalapa* where treatment with NPA delayed ethylene-induced abscission (Meir *et al.*, 2006). However, effects of NPA on senescence are consistent with those seen in *Iris*, another ethylene-insensitive flower (van Doorn *et al.*, 2013). Nevertheless, NPA did affect IAA levels differentially in the two genotypes, especially the levels of conjugated IAA, suggesting that auxin transport is required for determining levels of free auxin. The fall in expression of *APP1* in *Lilium* L.A. during senescence, but not in *L. longiflorum*, may suggest that auxin efflux is a factor in maintaining high levels of auxin in the latter. The distribution of auxin levels from the base to the tip of the petal fits with previous data showing higher levels at the base and is consistent with auxin transport in the petal during development. The effect of NPA suggests that the auxin transport is still active and is in the direction of the base to the tip since NPA treatment reduces auxin levels in the middle and tip sections and increases free auxin concentration at the base. The increase in auxin at the base with NPA treatment suggests a blockage of auxin transport due to the inhibitory effect of the NPA. This is probably into the base of the tepal from other floral organs, since during late tepal development and senescence, levels of auxin biosynthesis in the tepal itself are likely to be low (Aloni *et al.*, 2006). However, since this affects both lilies, the conclusion is that while auxin levels correlate with timing of abscission, transport of auxin does not.

## Conclusions

Despite their close genetic relationship, *L. longiflorum* and *Lilium* L.A. tepals age through different mechanisms, indicating that wilting and abscission strategies can differ in very closely related genotypes. The presence of a fully formed AZ in both genotypes suggests that the difference in abscission relates to the very last steps of AZ activation/completion. There is a clear correlation between auxin levels and abscission timing in relation to senescence markers. Furthermore, the role of auxin in abscission previously elucidated in *Arabidopsis* may involve the same mechanism through the *ARF* genes in this taxonomically unrelated group. Auxin transport does not affect senescence, and the effects of the auxin transport inhibitor NPA did not affect the two genotypes differentially, indicating that, although differing auxin levels may be responsible for the timing of tepal abscission, this is not due to differences in auxin transport during senescence.

## Supplementary data

Supplementary data are available at *JXB* online.

Supplementary Table S1. All primers used for PCR.

Supplementary Table S2. *L. longiflorum* contigs showing homology to *ARF* genes.

Supplementary Fig. S1. Total auxin levels as the sum of free and conjugated IAA.

Supplementary Fig. S2. Alignment of *ARF*-like lily sequences with nearest rice ORF match based on BLASTX homology.

Supplementary Fig. S3. Alignment of *APP1*-like lily sequence with the *Arabidopsis AtAPP1* gene (AT4G36760).

Supplementary Data

## Abbreviations:

ARF: auxin response factor
AZ: abscission zone
CB: closed bud
DW: dry weight
ES: early senescence
FS: full senescence
FW: fresh weight
FB: full bloom
IAA: indole-3-acetic acid
NPA: 1-*N*-naphthylphthalamic acid
SE: standard error.
