# Supplementary material for: Auxin involvement in tepal senescence and abscission in Lilium: a tale of two lilies
Source: J Exp Bot. 2014 Nov 24;66(3):945–56. doi: 10.1093/jxb/eru451 (PMC4321550; doi:10.1093/jxb/eru451)
Supplement: Supplementary Data [file supp_66_3_945__index.html]

Auxin involvement in tepal senescence and abscission in Lilium: a tale of two lilies — Auxin involvement in tepal senescence and abscission in Lilium: a tale of two lilies — Supplementary Data 

# Auxin involvement in tepal senescence and abscission in *Lilium*: a tale of two lilies

## Supplementary Data

Data files

**Files in this Data Supplement:**

- Supplementary Data - Supplementary Data
