# Supplementary material for: Auxin involvement in tepal senescence and abscission in Lilium: a tale of two lilies
Source: J Exp Bot. 2014 Nov 24;66(3):945–56. doi: 10.1093/jxb/eru451 (PMC4321550; doi:10.1093/jxb/eru451)
Supplement: Supplementary Data [file supp_eru451_jexbot135251_file001.pdf]

Supplementary Table S1: All primers used for PCR

primer	target	sequence
APP1dgF	Degenerate primers for APP1-like gene	CGACATGGTACWGGYCAT
APP1dgR		CATTCTCCAATCTWATNCCRAA
APP1sF	Specific primers for qPCR of *Lilium* APP1	TGTTCATGAAGGTCCCCATT
APP1sR		TTCCCGTCCTCATAGTAGCC
LlAUX1F	Primers designed to *L. longiflorum AUX1*-like contig	CCAAGTGCTACCAGTGCAAG
LlAUX1R		ACCAAACCCAAATTGCAAAC
LlARF7/19F	Primers designed to *L. longiflorum ARF7/19*-like contig	GACGGTGATCTAGGGAGCAA
LlARF7/19R		GCAGACGGTTTTCCAGGTTA
LlARF6/8F	Primers designed to *L. longiflorum ARF6/8*-like contig	ATGAGCTTGGGCAACTGTTT
LlARF6/8R		CAACCCCTTCTTTTCCCATT

Supplementary Table S2: *L. longiflorum* contigs showing homology to ARF genes

Lily contig	contig length	nearest rice gene (Wang, 2007)	nearest Arabidosis gene (Wang, 2007)	Arabidopsis expression during senescence (eFP browser)
651	3021	OsARF17	AtARF8/6	both down-regulated in older petals
1348	333	OsARF17	AtARF8/6	both down-regulated in older petals
1628	1400	OsARF1(?)	AtARF9/18/11	No change in expression
2468	2494	OsARF1	AtARF9/18/11	No change in expression
5803	264	OsARF21	AtARF7/19	up-regulated in old leaves, petals and sepals
6507	455	OsARF2	AtARF3/4	No change in expression
7111	974	OsARF19	AtARF7/19	up-regulated in old leaves, petals and sepals
8123	1078	OsARF14	AtARF3/4	No change in expression
9023	224	OsARF23	AtARF11	No change in expression
10581	260	OsARF17	AtARF8/6	both down-regulated in older petals
11452	254	OsARF2	AtARF3/4	No change in expression
11651	285	OsARF18	AtARF16	up-regulated in old leaves, and petals

Supplementary Fig. S1: Total auxin levels

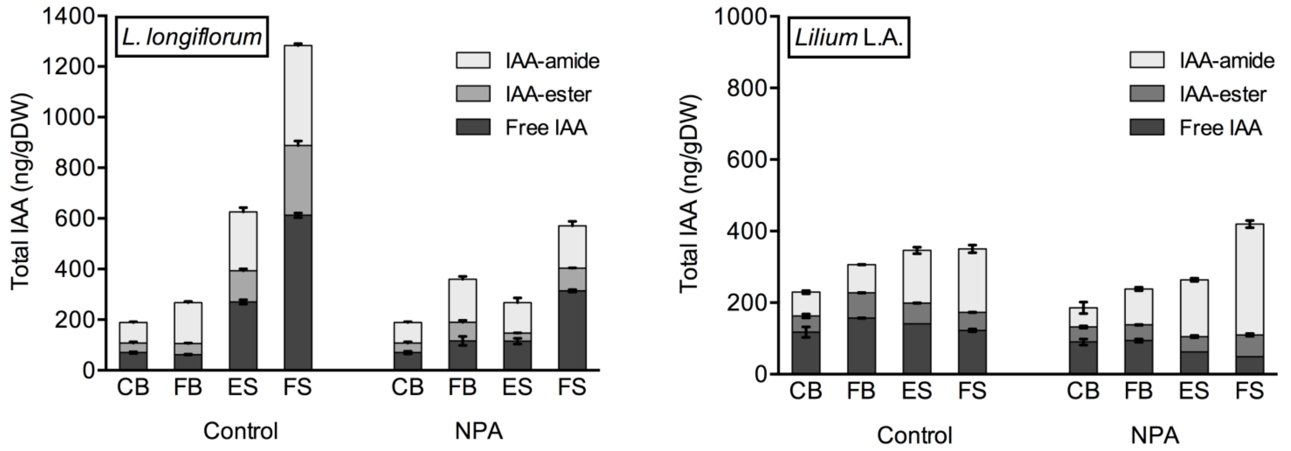

Supplementary Fig. S2: Alignment of *ARF*-like lily sequences with nearest rice ORF match based on BlastX homology

```
                                                  III
                     10        20        30        40        50        60
                 ....|....|....|....|....|....|....|....|....|....|....|....|
Q0D9R7.2-Os19    -LNRSSWPPAAPLKRMRTFTKVYKRGAVGRSIDMSQFSGYDELKHALARMFSIEGQLEER 59
7111             LLNTSSWAPAPPFQRMRTYTKVYKRGAVGRSIDMSRYSDYDELKHDLARMFSIEGQLEDR 60

                     70        80   IV   90       100       110       120
                 ....|....|....|....|....|....|....|....|....|....|....|....|
Q0D9R7.2-Os19    QRIGWKLVYKDHEDDILLLGDDPWEEFVGCVKCIRILSPQEVQQMSLEGCDLGNNIPPNQ 119
7111             QRVGWKLVYVDHENDILLVGDDPWEEFVNCVRFIKILSPQEVQQMRMDG-DLGSNI---Q 116

                    130       140       150       160       170       180
                 ....|....|....|....|....|....|....|....|....|....|....|....|
Q0D9R7.2-Os19    ACSSSDGGNAWRARCDQNSGNPSNGSYEQFE----------------------------- 150
7111             ACSSSDGGNAWRGQPEQISGNHSAGSYEHLELGYHRQLLLRLLFELLEVENLNLENRLLT 176
```

A: Alignment of contig 7111 with Os ARF19 (closest homologue to Arabidopsis ARF7 and ARF19 (conserved motifs III and IV are indicated in red)

```
Q653U3.1-Os17    TSQMEQLDSTAPSIPQ-SSALAPLPGRGCLVDQDGNSDPQNHLLFGVNIDSQSLLMQGGI 726
Lily_651         -TQVEQLCSPRTNISQPSVAQPPFSGRVCGHDQEGSTDPQSHLLFGVNIDSASLLAHHGV 822

                    850       860       870       880       890       900
                 ....|....|....|....|....|....|....|....|....|....|....|....|
Q653U3.1-Os17    PSLQGENDSTAIPYSTSNFLSPSQNDFPLDQTLSSADCLDESGYVPCSQNSDQVINRPPA 786
Lily_651         ---GDDIDSTSMPFPVTNFLGTMGTELPLNQAFTSSSGLEESGFMQSPDNLGQENPQN-G 878

                    910       920       930       940       950       960
                 ....|....|....|....|....|....|....|....|....|....|....|....|
Q653U3.1-Os17    TFVKVYKSGTYGRSLDITRFSSYHELRRELGRLFGLEGQLENPLRSGWQLVFVDREDDVL 846
Lily_651         TFVKVYKSGSFGRSLDISKFNSYSELRNELGQLFGLEGQLEDPKRSGWQLVFVDREDDVL 938
                               III                                IV

                    970       980       990      1000      1010      1020
                 ....|....|....|....|....|....|....|....|....|....|....|....|
Q653U3.1-Os17    LVGDDPWQEFVNSVSCIKILSPQEVQQMGKPFELLSSAPGKRLGSSCDDYVSRQESRSLS 906
Lily_651         LVGDDPWQEFVNNVWCIKILSPQQVLQMGKEGVDLQQNSMNLSSGITSGVDLQQNLMNLS 998
                            IV

                   1030
                 ....|....|.
Q653U3.1-Os17    TGIASVGSVEF 917
Lily_651         ----------- 998
```

B: Alignment of contig 651 with Os ARF17 (closest homologue to Arabidopsis ARF8 and ARF6 (conserved motifs III and IV are indicated in red)

Supplementary Fig. S3: Alignment of *APP1*-like lily sequence with Arabidopsis *AtAPP1* gene (AT4G36760)

```
                      10        20        30        40        50        60
              ....|....|....|....|....|....|....|....|....|....|....|....|
LLPP1-like    ------------------------------------------------------------ 1
AtAPP1        MSEILSSLRSLMASHSPPLDALVVPSEDYHQSEYVSARDKRREFVSGFSGSAGLALITKK 60

                      70        80        90       100       110       120
              ....|....|....|....|....|....|....|....|....|....|....|....|
LLPP1-like    ------------------------------------------------------------ 1
AtAPP1        EARLWTDGRYFLQALQQLSDEWTLMRMGEDPLVEVWMSDNLPEEANIGVDSWCVSVDTAN 120

                     130       140       150       160       170       180
              ....|....|....|....|....|....|....|....|....|....|....|....|
LLPP1-like    ------------------------------------------------------------ 1
AtAPP1        RWGKSFAKKNQKLITTTTDLVDEVWKSRPPSEMSPVVVHPLEFAGRSVSHKFEDLRAKLK 180

                     190       200       210       220       230       240
              ....|....|....|....|....|....|....|....|....|....|....|....|
LLPP1-like    ------------------------------------------------------------ 1
AtAPP1        QEGARGLVIAALDEVAWLYNIRGTDVAYCPVVHAFAILTTDSAFLYVDKKKVSDEANSYF 240

                     250       260       270       280       290       300
              ....|....|....|....|....|....|....|....|....|....|....|....|
LLPP1-like    ------------------------------------------------------------ 1
AtAPP1        NGLGVEVREYTDVISDVALLASDRLISSFASKTVQHEAAKDMEIDSDQPDRLWVDPASCC 300

                     310       320       330       340       350       360
              ....|....|....|....|....|....|....|....|....|....|....|....|
LLPP1-like    ------------------------------------------------------------ 1
AtAPP1        YALYSKLDAEKVLLQPSPISLSKALKNPVELEGIKNAHVRDGAAVVQYLVWLDNQMQELY 360

                     370       380       390       400       410       420
              ....|....|....|....|....|....|....|....|....|....|....|....|
LLPP1-like    ------------------------------------------------------------ 1
AtAPP1        GASGYFLEAEASKKKPSETSKLTEVTVSDKLESLRASKEHFRGLSFPTISSVGSNAAVIH 420

                     430       440       450       460       470       480
              ....|....|....|....|....|....|....|....|....|....|....|....|
LLPP1-like    ------------------------------------------------------------ 1
AtAPP1        YSPEPEACAEMDPDKIYLCDSGAQYLDGTTDITRTVHFGKPSAHEKECYTAVFKGHVALG 480

                     490       500       510       520       530       540
              ....|....|....|....|....|....|....|....|....|....|....|....|
LLPP1-like    -----------------------------HGTGHGIGSYLNVHEGPHLISFRPHARNVPL 31
AtAPP1        NARFPKGTNGYTLDILARAPLWKYGLDYRHGTGHGVGSYLCVHEGPHQVSFRPSARNVPL 540

                     550       560       570       580       590       600
              ....|....|....|....|....|....|....|....|....|....|....|....|
LLPP1-like    QASMTVTDEPGYYEDGNFGIRLEN------------------------------------ 55
AtAPP1        QATMTVTDEPGYYEDGNFGIRLENVLVVNDAETEFNFGDKGYLQFEHITWAPYQVKLIDL 600

                     610       620       630       640
              ....|....|....|....|....|....|....|....|....|
LLPP1-like    --------------------------------------------- 55
AtAPP1        DELTREEIDWLNTYHSKCKDILAPFMNQTEMEWLKKATEPVSVSA 645
```
